# Case Report: Supramalleolar osteotomy to preserve joint function and delay the development of Charcot neuroarthropathy of the ankle

**DOI:** 10.3389/fsurg.2023.1292120

**Published:** 2023-11-20

**Authors:** Baozhou Zhang, Wenjing Li, Ying Li, Hui Du, Yong Wu

**Affiliations:** ^1^ Department of Foot and Ankle Surgery, Beijing JiShuiTan Hospital, The Fourth Clinical College of Peking University, Beijing, China; ^2^ Department of Foot and Ankle Surgery, Beijing JiShuiTan Hospital, Capital Medical University, Beijing, China

**Keywords:** supramalleolar osteotomy, Charcot neuroarthropathy, ankle function, quality of life, Charcot arthritis

## Abstract

**Background:**

Charcot neuroarthropathy (CN) is a severe disease that primarily affects the foot and ankle. Conservative treatment with total contact casts is suitable for early stages, but surgery is necessary for complications such as ulceration, malalignment, infection, or severe pain. The ankle instability caused by excessive axial load may require arthrodesis. However, preserving joint function in young patient can significantly enhance the quality of life.

**Case report:**

A 33-year-old woman underwent open reduction and internal fixation after the right tibia and fibula fractures following a fall while walking. She developed severe pain and deformity in her right ankle after full weightbearing. After further evaluation, she was diagnosed with Charcot neuroarthropathy (CN) of the right ankle. The patient declined arthrodesis and opted for a supramalleolar osteotomy (SMO) instead 18 months after the initial surgery. The SMO procedure involved correcting the hindfoot malalignment through osteotomy and fixation. Although she experienced skin necrosis, the patient eventually achieved satisfactory outcomes with improvements in pain, deformity, and functionality of the ankle. Radiographic measurements showed positive realignment, and the patient reported a significant improvement in her quality of life at the final follow-up.

**Conclusions:**

The SMO procedure could potentially be considered as an option to preserve ankle function and delay the disease development of CN for young patients. The restored foot stability and hindfoot alignment can help improve patients' quality of life.

## Introduction

Charcot neuroarthropathy (CN) is a devastating disease that profoundly affects the musculoskeletal system (1, 2). It is commonly associated with diabetic neuropathy, and may be involved with alcoholism, syringomyelia, rheumatoid arthritis, multiple sclerosis, and traumatic injury (2–5). The region encompassing the foot and ankle tends to be most susceptible, often presenting with a red, hot, and swollen foot, accompanied by pain or not (1, 6, 7). The collapse, fractures, and joint destruction that occur in the foot and ankle can result in the inability to bear weight, leading to a substantial deterioration in the patient's quality of life (1, 4, 8).

The Modified Eichenholtz Classification provides a comprehensive delineates for comprehending the progression of CN, dividing it into distinct stages: development (fragmentation), coalescence, and reconstruction and reconstitution (9). Drawing from the Brodsky classification (10), the midfoot emerges as the most prevalent region of affliction, with the tarsometatarsal and talonavicular joints frequently ensnared. Lesions in the hindfoot precipitate an alarming instability in the joint, potentially leading to the breakdown of the proximal ankle. The excessive axial load exerted on the ankle causes significant damage and gives rise to catastrophic deformities. Treatment strategies vary depending on the stage and severity of the condition. The ultimate objective in managing CN of the foot and ankle is to achieve a plantar-grade foot without ulcerations (1, 10).

Conservative treatment is typically appropriate for the early stages of foot and ankle CN. The gold standard in conservative management is the use of a total contact cast (TCC) (7, 8, 11). Nevertheless, surgical intervention becomes imperative when recurrent ulceration, severe axial malalignment, deep infection, or debilitating pain occur (1, 12). Excessive axial load has the potential to induce hindfoot malalignment, leading to the manifestation of ankle deformities. In such circumstances, it is advisable to consider employing arthrodesis as an intervention to rectify the ankle deformities (10, 13, 14). Therefore, it is worth investigating whether improving ankle alignment can delay the CN progression and preserve ankle function. In current case report, we present a captivating case where supramalleolar osteotomy (SMO) was employed to effectively postpone the development of ankle CN and preserve ankle function.

## Case description

**Figure 1:**
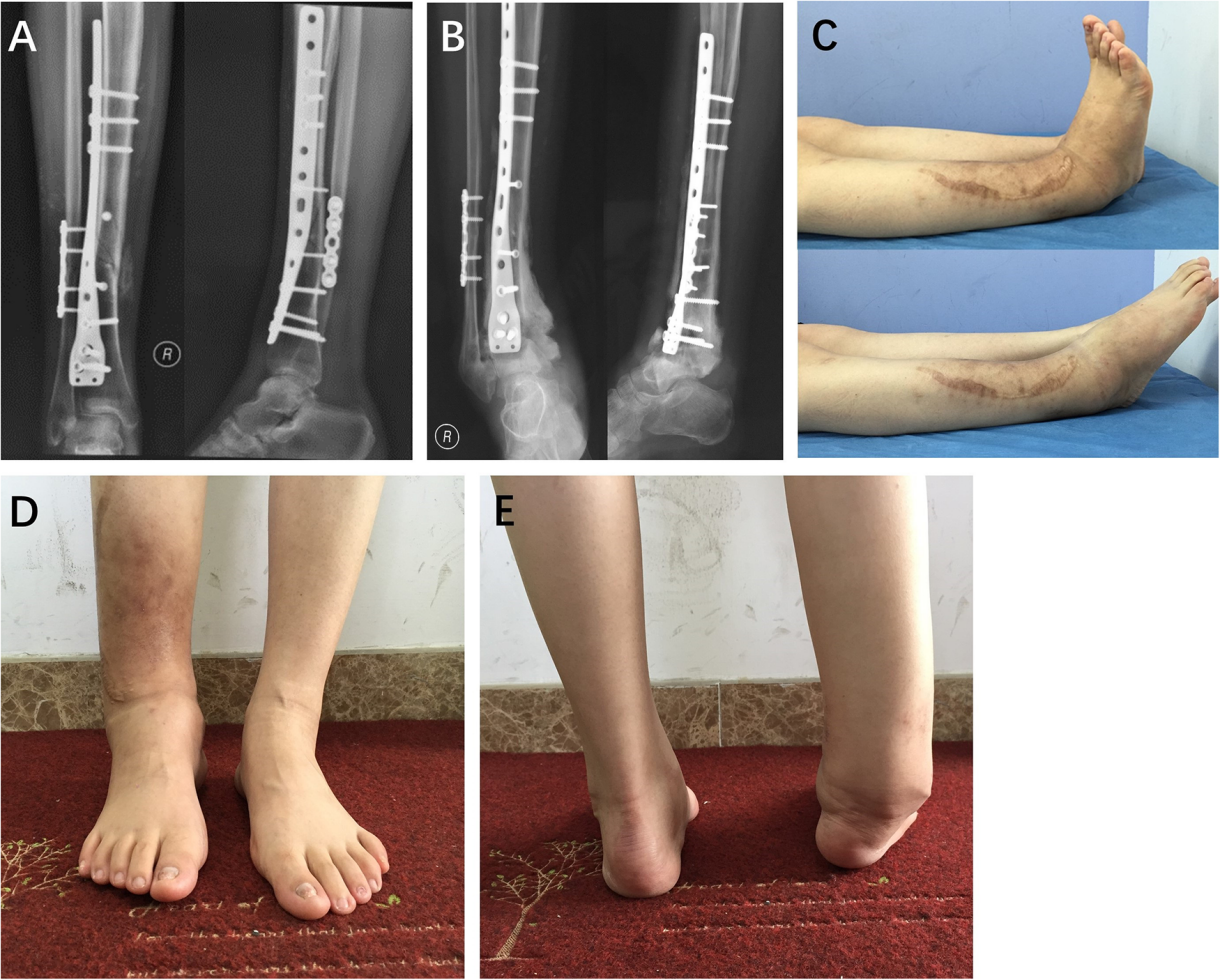
A 33-year-old woman underwent open reduction and internal fixation (**A**,**B**) following fractures of the right tibia and fibula caused by a fall. The range of motion of the right ankle is depicted in panel (**C**). The deformity of the right ankle is illustrated in panels (**D**,**E**).

In December 2014, a 33-year-old woman sustained fractures of the tibia and fibula following a fall while walking. The open reduction and internal fixation was performed to fix tibia and fibula fractures at other hospital (Figure 1). The patient began partial weightbearing 9 months post-surgery and progressed to full weightbearing at 12 months. However, after achieving full weightbearing, she experienced severe pain and deformity in her right ankle, leading her to seek further diagnosis and treatment at our hospital. Physical examination revealed mild swelling, a varus deformity of the right ankle, and an unsteady gait. No ulcers were observed in the distal extremity. Dorsiflexion of the right ankle was limited to 0 degrees. Radiographic imaging revealed destruction and varus deformity of the distal tibia, along with anterior subluxation of the talus. Routine preoperative laboratory examinations revealed no noteworthy abnormalities. The patient has been afflicted with type 1 diabetes since the age of 11. The patient's preoperative fasting blood glucose was controlled at around 5 mmol/L. Based on the above information, the patient was diagnosed with CN of the right ankle (Modified Eichenholtz Classification Stage 3).

**Figure 2:**
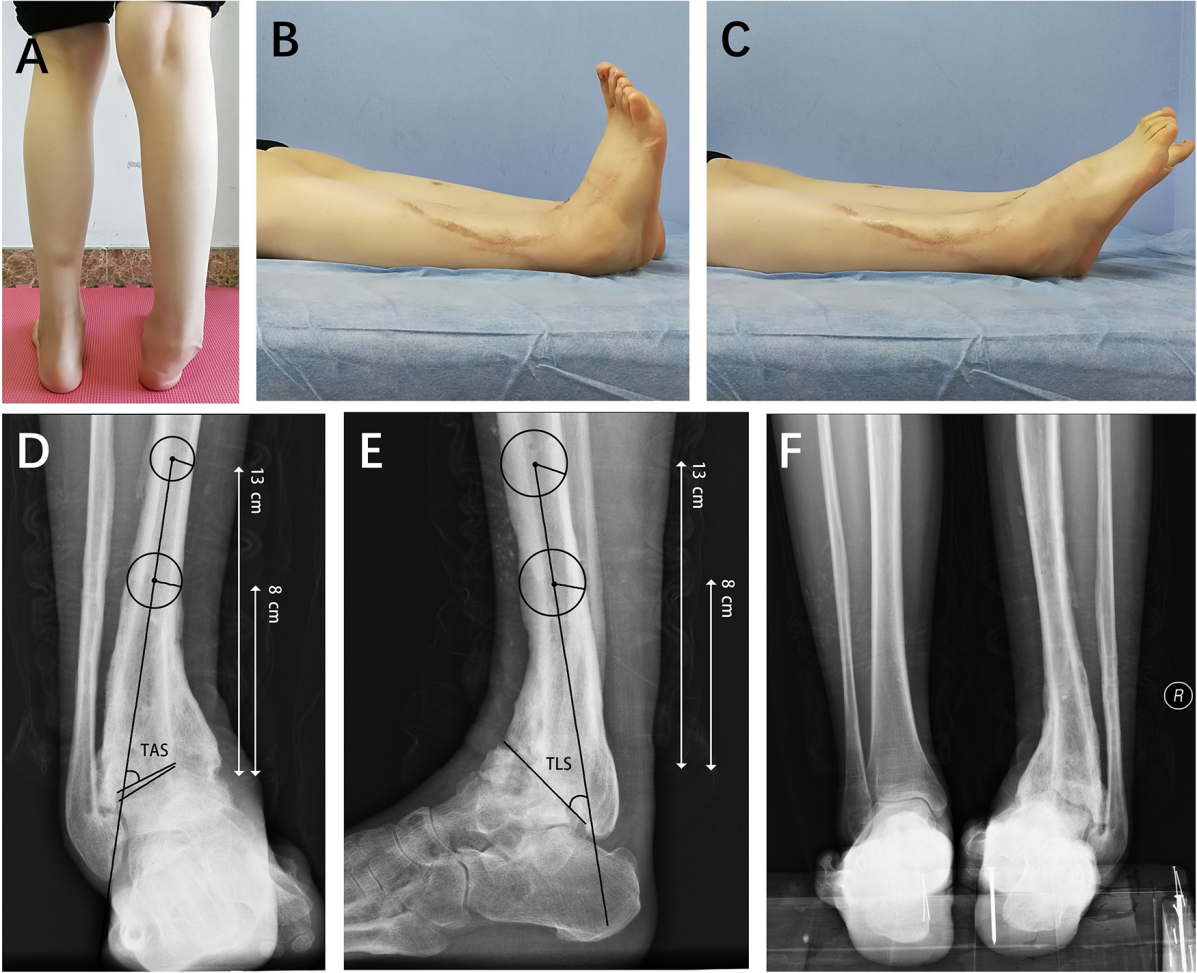
The internal fixation devices were removed 18 months after the initial surgery. The deformity of the right ankle is shown in panel (**A**), and the range of motion is displayed in panel (**B**,**C**). Radiographic measurements revealed that the angles of the tibial articular surface (TAS) and tibial lateral surface (TLS) were 54 and 50 degrees, respectively (**D**,**E**). Panel (**F**) depicts the hindfoot malalignment.

**Figure 3:**
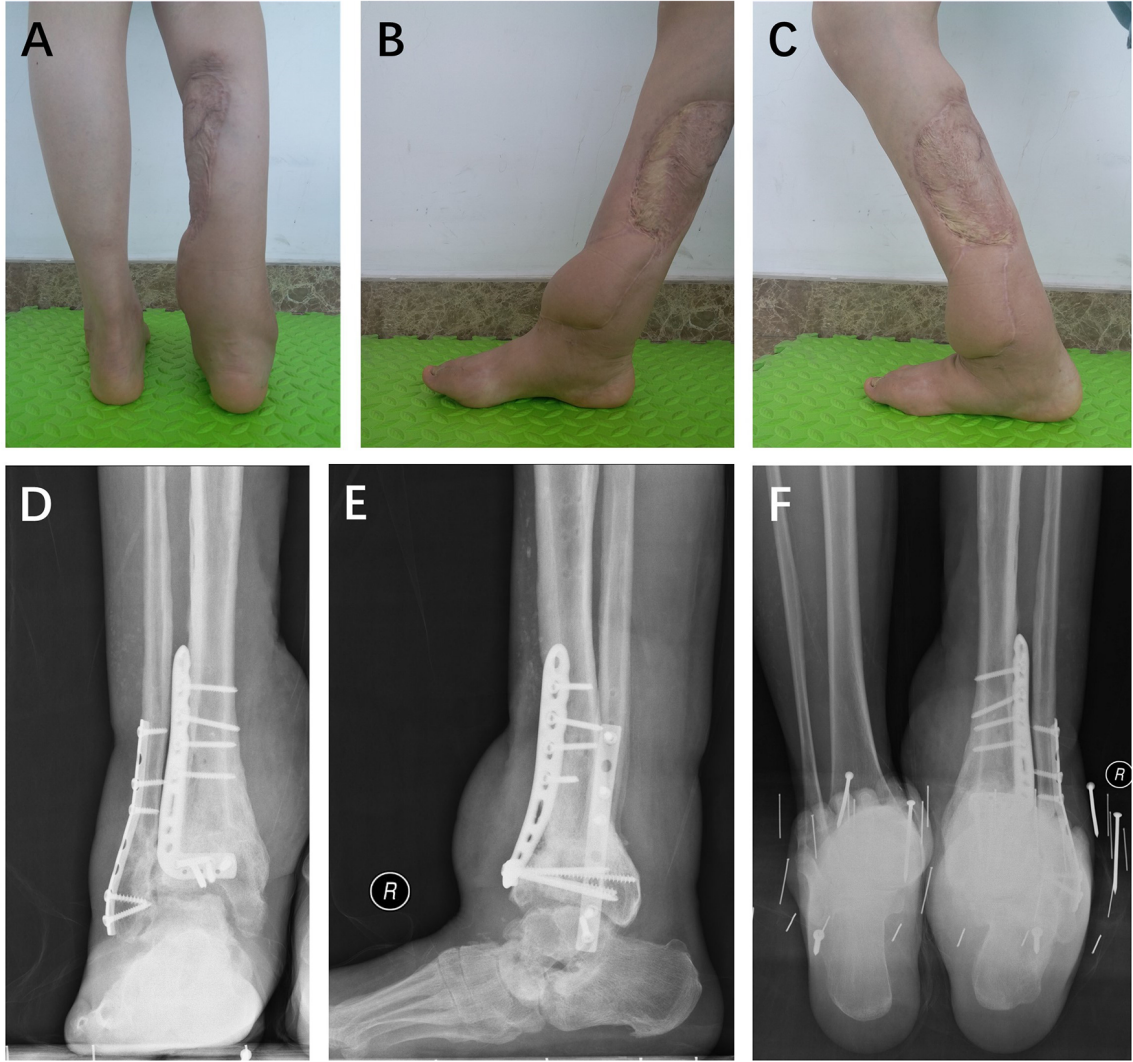
At the 18-month follow-up after the supramalleolar osteotomy, the patient displayed satisfactory hindfoot alignment (**A**) and range of motion (**B**,**C**). Charcot arthritis was detected at the Chopart joint (**D**,**E**), although no apparent symptoms were present. Radiographic images demonstrated the satisfactory hindfoot alignment (**F**).

The internal fixation devices were removed 18 months after the initial surgery (Figure 2), but the patient continued to experience unresolved pain, swelling, and deformity. The Visual Analog Scale (VAS) score for pain was rated at 4. Radiographic measurements revealed that the angles of the tibial articular surface (TAS) and tibial lateral surface (TLS) were 54 and 50 degrees, respectively. The American Orthopaedic Foot and Ankle Society (AOFAS) ankle-hindfoot score (15) was 50. Despite recommending arthrodesis, the patient strongly declined and sought further intervention. Considering the hindfoot malalignment and excessive axial load, we made the decision to proceed with a SMO. The procedure was performed by the senior author (Ying Li). An anterior approach was used to carry out a medial-anterior opening-wedge osteotomy of the distal tibia. The osteotomy plane was positioned 2 cm above the ankle. The bone was then cut from an anterior-medial-superior to posterior-lateral-inferior direction. Subsequently, an osteotome was employed to complete the tibia osteotomy. A wedge-shaped piece of bone, sourced from the patient's iliac bone, was inserted to fill the tibia osteotomy site. Following correction in both the coronal and sagittal planes, an anterior tibial plate and a 1/3 tubular plate on the lateral aspect of the fibula were used for fixation. Postoperative radiographs demonstrated satisfactory alignment and positioning of the ankle. The subsequent management involved 3 weeks of non-weightbearing with a posterior splint. Unfortunately, skin necrosis occurred three weeks after the surgery, and although intravenous antibiotics of Ceftazidime were administered, no improvement in the condition was observed. A sural flap procedure was performed, and the wound site healed without complications. Full weightbearing commenced 12 weeks postoperatively, and at the 6-month follow-up, the patient exhibited an adequate range of motion. The osteotomy union was observed after the SMO. Charcot arthritis was detected at the Chopart joint 18 months after the SMO, but no apparent symptoms were present, and an increase in dorsiflexion was noted (Figure 3).

**Figure 4:**
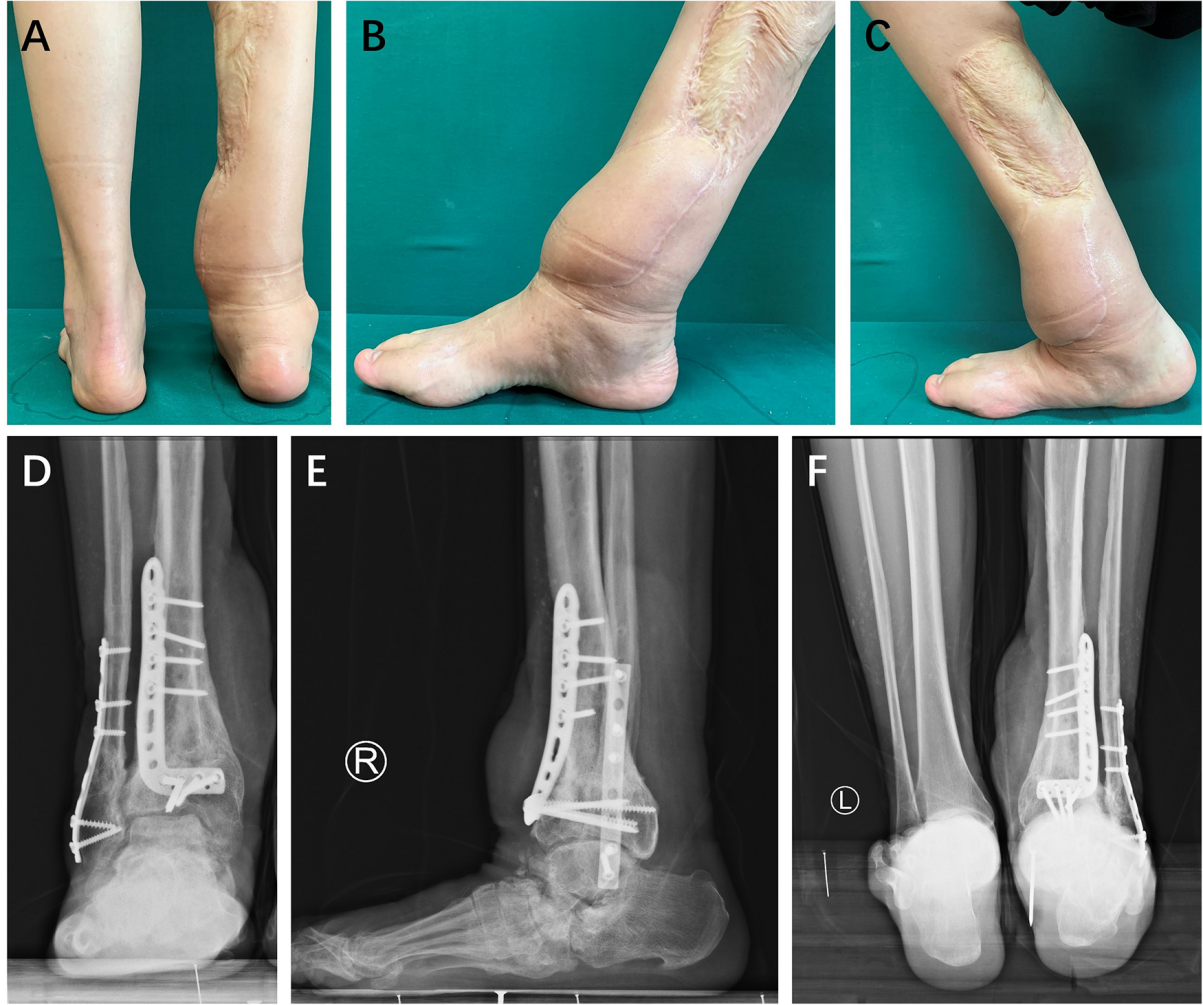
Three years after the supramalleolar osteotomy, the patient showed satisfactory hindfoot alignment (**A**) and an adequate range of motion (**B**,**C**). Radiographic images revealed the presence of osteophytes anterior to the talus (**D**,**E**). The hindfoot alignment remained satisfactory (**F**).

Three years after the SMO procedure, the patient reported experiencing mild anterior pain and swelling in the right ankle during prolonged walking. Radiographic imaging revealed the presence of osteophytes anterior to the talus (Figure 4). In order to address this issue, arthroscopic exostectomy was performed through anteromedial and anterolateral portals. During the arthroscopy, the osteophytes were visualized and removed. Importantly, the integrity of the ankle cartilage showed significant improvement.

Upon reaching the final follow-up, notable improvements were observed in the pain, deformity, and function of the right ankle. The VAS pain score decreased from 4 to 1, indicating a significant reduction in pain. Furthermore, the TAS angle, which was originally at 54 degrees, was corrected to 85 degrees, while the TLS angle improved from 36 to 82 degrees. These radiographic improvements indicate a favorable realignment of the ankle joint. In terms of functional assessment, the AOFAS ankle-hindfoot score increased from 50 to 83. No significant progression was found regarding to Charcot arthritis at the Chopart joint. No ulceration was observed on the foot and ankle. This indicates a substantial enhancement in joint function. Most importantly, the patient expressed satisfaction with their improved quality of life following the intervention.

## Discussion

CN is a distressing disease that greatly impacts patients' quality of life (16–18). In particular, when CN affects the foot and ankle, it frequently occurs in conjunction with diabetes, resulting in an inability to maintain weight-bearing status and body balance (10). In order to enhance patient prognosis, proactive intervention assumes a critical role. The primary aim of surgical intervention is to restore proper alignment and stability, thereby preventing the recurrence of ulceration and the potential amputation (1, 8, 10).

The chosen treatment strategy varies based on the stage and severity of the CN condition. In line with the classification of Charcot, conservative methods are typically suitable for patients in the early stages (Eichenholtz stages 0, I, and II). These methods include reducing physical activity and off-loading the affected extremity (6, 7). It is recommended to off-load for a duration of 2–6 months or even longer (1), as this reduces the likelihood of recurrence. A more prolonged period of immobilization can assist in alleviating acute CN symptoms and minimizing the risk of fracture or deformity. TCC employment is considered the gold standard in conservative management (7, 8, 11). The use of an ankle-foot orthosis is commonly employed to reduce the patient's activity level (1). Current research suggests that using a non-removable immobilizer for protected weight-bearing, coupled with frequent follow-up, demonstrates a safe and effective treatment strategy for acute CN (1).

When non-surgical treatments prove unsuccessful or unfeasible, surgical intervention becomes a necessary course of action. Indications for surgery include a destructive foot and ankle architecture, recurring ulceration, severe axial malalignment, deep infection, or debilitating pain (1, 12). The specific surgical approach depends on the progression of CN, encompassing techniques such as exostectomy, osteotomy, Achilles tendon lengthening, and arthrodesis (19, 20). Exostectomy is an ideal method for stabilizing foot with ulcers caused by bone protrusions (1). However, in cases where the foot is unstable and experiences devastating deformities due to high stress from axial load, arthrodesis becomes necessary to restore alignment and stability (13, 14). Internal fixation, external fixation, or a combination application are utilized for arthrodesis in the management of CN in the foot and ankle (10). Many literature recommends arthrodesis in order to correct alignment and achieve satisfactory clinical outcomes (1, 10, 13, 14). However, it is crucial to acknowledge that arthrodesis is a joint-sacrificing procedure and therefore may not provide lifelong relief. Particularly for young patients, they require adequate range of motion in the ankle joint for massive activity. To ensure a satisfactory long-term result and improve quality of life, it is prudent to consider a joint-sparing procedure as a potential option.

In present case, the patient expressed a strong desire to preserve ankle function and declined arthrodesis surgery. Therefore, we opted for an adventurous approach and performed a SMO for the patient with severe right ankle joint deformity and CN. To our knowledge, there have been no previous documented cases demonstrating the use of SMO for correcting deformities associated with ankle CN. Typically, SMO is not recommended for patients with CN. However, remarkably, the current case report presents the first successful implementation of SMO in patients with ankle CN, resulting in satisfactory clinical outcomes. The fragments of osteotomy healed successfully after SMO. Surprisingly, the patient manifested good ankle mobility and stability with no notable progression of CN even in the almost five-year period following the SMO. The VAS score decreased from 4 to 1, and the AOFAS ankle-hindfoot score increased from 50 to 83. The TAS and TLS angles were corrected from 54 and 36 degrees to 85 and 82 degrees, respectively. We attribute these remarkable outcomes to the correction of hindfoot alignment. The alignment of the hindfoot is crucial for the stability of the foot and ankle. Eighteen months following the SMO procedure, Charcot arthritis was detected at the Chopart joint, yet no noticeable symptoms were observed. Despite ankle function maintained, the Charcot's arthritis in the midfoot was found. Performing hindfoot arthrodesis would subject the midfoot to increased stress, potentially leading to accelerated and more severe joint degeneration in theory. Consequently, our conviction in the ability of SMO to delay the progression of Charcot neuroarthropathy in young patients has grown stronger. However, it is important to acknowledge that arthrodesis remains a highly probable treatment option for the patient in the future. Correcting hindfoot alignment and delayed arthrodesis could be considered as an alternative for young patients with hindfoot malalignment in order to improve their quality of life.

Due to the limited availability of data, it is important to note that the conclusions drawn from this isolated case may not be entirely conclusive. The presentation and progression of CN can vary greatly among patients, leading to heterogeneity in treatment options. It is crucial to carefully consider individual patient factors when determining the most appropriate surgical strategy. In this case report, we aim to provide potential treatment options for patients who exhibit similar symptoms and progression as the case presented.

## Conclusion

The SMO procedure could potentially be considered as an option to preserve ankle function and delay the disease development of CN for young patients. The restored foot stability and hindfoot alignment can help improve patients' quality of life.

## Data Availability

The raw data supporting the conclusions of this article will be made available by the authors, without undue reservation.
